# Unveiling immune tolerance pathways in preeclampsia placenta: implications for molecular targets and discovery of potential biomarkers

**DOI:** 10.3389/fendo.2024.1385154

**Published:** 2024-06-04

**Authors:** Yantuanjin Ma, Xingli Deng, Ru Shen, Hongqing Zhang, Yuan Qian

**Affiliations:** ^1^ Institute of Biomedical Engineering, Kunming Medical University, Kunming, China; ^2^ Kunming Medical University, Kunming, China; ^3^ Department of Neurosurgery, The First Affiliated Hospital of Kunming Medical University, Kunming, China; ^4^ Clinical Medical Research Center for Obstetrics and Gynecology (Yunnan Joint Key Laboratory), Kunming City of Maternal and Child Health Hospital, Kunming City of Women and Children, Kunming, China

**Keywords:** preeclampsia, placenta, bioinformatics, immunology, diagnostic markers

## Abstract

During pregnancy, there is a link between disruption of maternal immune tolerance and preeclampsia, but the molecular mechanisms that regulate maternal and fetal immune tolerance remain unclear. This study employs bioinformatics to identify new markers related to placental immune tolerance and explore their potential role in predicting preeclampsia. Analyzing preeclampsia-related gene expression profiles in the Gene Expression Omnibus (GEO) dataset reveals 211 differentially expressed genes (DEGs) in the placenta, mainly influencing immune cell differentiation and response pathways. Employing weighted gene co-expression network analysis (WGCNA) and lasso regression, four potential target genes (ANKRD37, CRH, LEP, SIGLEC6) are identified for potential prediction of preeclampsia. Validation using the GSE4707 dataset confirmed the diagnostic and predictive potential of these candidate genes. RT-qPCR verified up-regulation in the placenta, while ELISA showed their correlation with immune tolerance factors associated with placental immune tolerance. As a result of this study, identifies potential biomarkers associated with placental immunity and contributes to understanding the molecular mechanism of preeclampsia.

## Introduction

Preeclampsia is characterized by high blood pressure, with or without proteinuria, occurring during pregnancy, typically after 20 weeks of gestation (1). Worldwide, it continues to be one of the leading causes of maternal and perinatal mortality and morbidity (2, 3). According to the World Health Organization (WHO), preeclampsia contributes to a significant number of maternal deaths and adverse perinatal outcomes globally each year, posing a substantial public health concern (4). Despite advances in obstetric care, the pathophysiological mechanisms of preeclampsia remain poorly understood, making it difficult to develop effective preventive and therapeutic measures.

Preeclampsia’s pathogenesis is multifaceted and currently under investigation. Endothelial dysfunction, immune maladaptation, abnormal placental development, and vascular injury are significant factors contributing to preeclampsia (4–7). Inflammatory and anti-inflammatory mediators are imbalanced in immune dysregulation, and this is associated with the pathogenesis of preeclampsia, although the precise immunological events remain unclear (8, 9). Furthermore, abnormal placental perfusion resulting from impaired trophoblast invasion and spiral artery remodeling plays a crucial role in the development of the disorder (10).

Studies have demonstrated the involvement of various immune cells, including T cells, B cells, natural killer cells, and macrophages, in the immunological imbalance at the maternal-fetal interface in preeclampsia (11, 12). These immune cells are believed to be the principal contributors to the systemic inflammatory response and endothelial dysfunction. Additionally, the abnormal release of cytokines and other inflammatory markers in the placental and maternal circulation is considered to promote vascular endothelial cell injury, ultimately leading to the clinical manifestations of preeclampsia (13, 14).

Understanding the intricate interplay between immune dysregulation and placental abnormalities in preeclampsia is crucial for the development of targeted therapeutic strategies and improved clinical management. We used a bioinformatics approach to analyze gene expression profiles associated with preeclampsia in order to identify key immune pathways and potential biomarkers relevant to the pathogenesis and clinical manifestations of preeclampsia. Preeclampsia’s complex immunological and vascular mechanisms are revealed in these findings, and they may help develop new diagnostic tools and therapeutic interventions to reduce its devastating effects.

## Materials and methods

### Participants and placenta collection

The study involved pregnant women registered at the first affiliated Hospital of Kunming Medical University, comprising 6 cases in the preeclampsia group and 6 cases in the healthy control group. Diagnostic criteria for preeclampsia followed the guidelines of the American College of Obstetricians and Gynecologists (ACOG), requiring blood pressure readings over 140/90 mm Hg, with or without proteinuria. Exclusion criteria comprised chronic hypertension, illicit drug use or smoking, diabetes, abortion, kidney and cardiovascular disease, chorioamnionitis, and autoimmune diseases (whether taking medication or low-dose aspirin). Healthy pregnant women without a history of delivery complications constituted the control group (matched for pregnancy and delivery times, as well as delivery methods).

Clinical characteristics of the patients were documented, with details presented in **Supplementary Table 1**. Placental tissue collection occurred promptly post-delivery. Tissue biopsies, approximately 1.0~3cm in size, were obtained from the placental center (avoiding vascular and/or calcium deposition) on both the fetal and maternal sides, then stored at -80°C for subsequent analysis. Informed consent was obtained from all participants, and approval for this study was granted by the First Affiliated Hospital of Kunming Medical University (Ethical approval number 31-2, 2022).

### Microarray dataset collection

**Figure 1:**
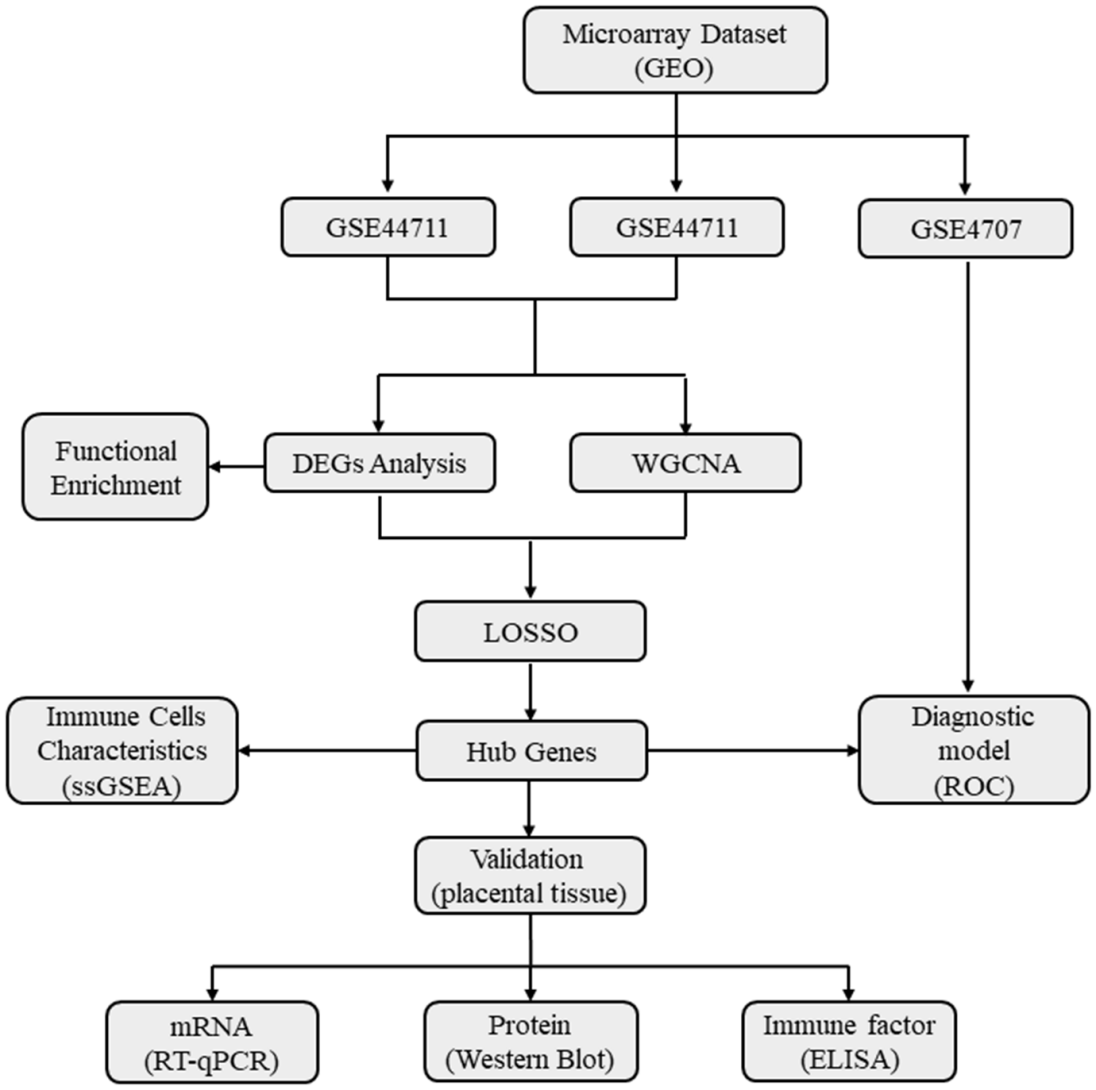
A flow diagram showing the research methodologies employed in this study. GEO, Gene Expression Omnibus Database; WGCNA, Weighted gene co-expression network analysis; DEGs, Differentially expressed genes; Functional enrichment includes Gene ontology (GO), KEGG, Kyoto Encyclopedia of Genes and Genomes; GSEA, Gene set enrichment analysis; ssGSEA, Single sample gene set enrichment analysis; LASSO, Least absolute shrinkage and selection operator; ROC, Receiver operating characteristic curve.

The placental transcriptome dataset was obtained from the Gene Expression Omnibus (GEO) database, comprising three sets of expression profiles (GSE44711, GSE66273, and GSE4707) and corresponding platform information (Platforms GPL10558, GPL4133, and GPL1708). The research methodology employed in this study is illustrated in **Figure 1**.

### Differentially expressed genes analysis

The R package ‘LIMMA’ was used for differential analysis. DEGs in GSE44711 and GSE66273 were identified with the criteria logFC > 1 and p < 0.05. The volcano plot and heat map of DEGs were simultaneously generated using the ‘ggplot2’ and ‘pheatmap’ packages.

### Functional enrichment analysis of DEGs

For a deeper understanding of DEGs’ role in preeclampsia, the Bioconductor package ‘clusterProfiler’ in R was used to conduct Gene Ontology (GO) analysis and Kyoto Encyclopedia of Genes and Genomes (KEGG) enrichment analysis of DEGs. Additionally, the Immune Signatures Database was used for gene set enrichment analysis (GSEA) of transcriptome changes. The aim was to elucidate the immunological functions of DEGs.

### Weighted gene co-expression network analysis and module gene screening

Employing the WGCNA algorithm, a co-expression network of placental samples was constructed to pinpoint gene modules linked to preeclampsia. Assessment of missing data points in the samples preceded clustering. The ‘pickSoftThreshold’ function was then utilized to determine the suitable soft threshold, facilitating the establishment of a scale-free network with biological significance. From the adjacency matrix, the topological overlap matrix was derived. The dynamic tree cutting algorithm, with parameters including minimum module size =30, deep division =2, and combined cutting height =0.25, was employed to pinpoint gene modules. Hierarchical clustering was utilized to create a tree diagram. Gene significance (GS) and module membership (MM) were calculated, along with modules associated with clinical features, enabling visualization of feature gene networks. The expression profile of each gene was summarized by module genes, and the correlation between module genes and traits was computed. Modules exhibiting the highest correlation and intersection of DEGs were selected for further analysis.

### Screening and verification of characteristic genes of preeclampsia

Utilizing the test datasets GSE44711 and GSE66273, genes intersecting between modules exhibiting the highest correlation from DEG and WGCNA analyses were identified for investigation. The Least Absolute Shrinkage and Selection Operator (LASSO), an algorithm for regression analysis utilized for variable selection to prevent overfitting, was implemented via the ‘glmnet’ package. ROC curves were constructed using the ‘pROC’ package to assess diagnostic performance. The Area Under the ROC Curve (AUC) was computed to ascertain the accuracy and efficacy of candidate genes. Logistic regression analysis, integrating the expression levels of key genes, was conducted through the RMS software package to formulate a nomogram model for preeclampsia diagnosis. To assess the model’s predictive ability, the C index was computed, and a calibration curve was generated. Lastly, the testing datasets GSE4707 and clinical samples were chosen to validate the expression of characteristic genes. Visualization was accomplished using the ‘boxplot’ and ‘pROC’ packages in R.

### Characteristics of placental immune cells

To evaluate the immunological characteristics of placental samples from preeclampsia, 28 types of inflammatory gene sets were analyzed through single-sample gene set enrichment analysis (ssGSEA) using the GSVA R software package. Lastly, we constructed correlation matrices for all 28 types of immune cells and correlation coefficients between characteristic genes and immune cells displaying significant differences in expression.

### RNA extraction and quantitative real-time PCR

Total RNA was extracted from placental tissue by Trizol reagent (Thermo Fisher Scientific, USA). A reverse transcription kit (TaKaRa, Japan) was used to reverse transcribe the mRNA into cDNA. Using the SYBR Green PCR master mix (Roche, Germany) and appropriate primers listed in **Table 1**, quantitative real-time PCR was conducted on an ABI Q6 instrument (Applied Biosystems, USA). Based on a reference of β-actin, it was calculated the relative expression levels of each characteristic gene.

**Table 1:** Candidate gene primer sequence.

Gene	primer sequence	
β-Actin	Forward	AGAGCTACGAGCTGCCTGAC
	Reverse	AGCACTGTGTTGGCGTACAG
ANKRD37	Forward	AGCAAGTGAACACCCTGACA
	Reverse	CCCACGTGACATCAGCACTT
CRH	Forward	TGGGGAACCTCAACAAGAGC
	Reverse	CAGCAACACGCGGAAAAAGT
LEP	Forward	CATTTCACACACGCAGTCAGT
	Reverse	TGGAAGGCATACTGGTGAGG
SIGLEC6	Forward	TTCCCCTTCTCTGCTCATGC
	Reverse	CATAGTACGAGGCTGCCCAC

### Western blot analysis

RIPA lysis buffer supplemented with PMSF was used to extract total protein from placental tissue, and 50 μg of protein were loaded per lane. SDS-PAGE gels were used to separate the proteins, they were transferred to a PVDF membrane, and they were blocked with 5% milk for 60 minutes at room temperature. Western blot analysis was performed with Anti-CRH antibody (Abcam, USA), Anti-LEP antibody (Abcam, USA), Anti-ANKRD37 antibody (Thermo Fisher Scientific, USA), and SIGLEC6 antibody (Abcam, USA), followed by overnight incubation at 4°C. Secondary antibodies conjugated to HRP bound to the primary antibodies. The image was visualized using a chemiluminescence detection imaging system. Using β-actin as an internal reference, the relative quantities of proteins were determined for each group.

### Immune factors detection

Interferon-γ (IFN-γ), transforming growth factor-β (TGF-β), interleukin-10 (IL-10), and interleukin-4 (IL-4) were detected in placental tissue using an enzyme-linked immunosorbent assay (ELISA) kit (Elabscience, China). Standard curves were constructed using dilutions of cytokine standards provided in the kit to calculate the concentrations of cytokines. Triplicate measurements were taken for all cytokines.

### Statistical analysis

Analyzing the experimental data was carried out using SPSS 22.0. The measured data are presented as mean ± standard deviation. In order to compare the data between the two groups, the t-test was conducted, with statistical significance defined as P < 0.05.

## Result

### DEGs in placenta of preeclampsia

**Figure 2:**
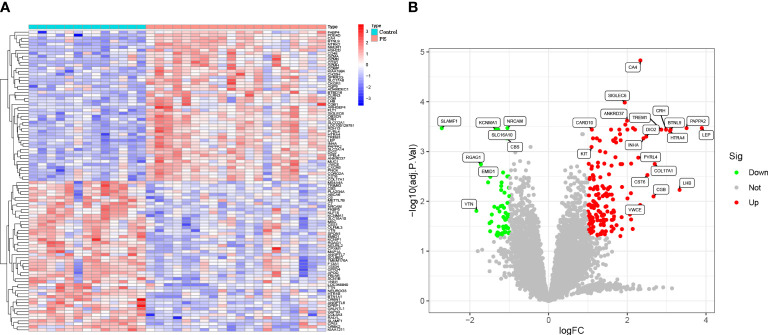
Identification of DEGs in the GSE44711 dataset and GSE66273 dataset of Preeclampsia. **(A)** Heatmap to visualize the top 100 DEGs; **(B)** A volcano plot of all DEGs.

Combining GSE44711 and GSE66273 datasets as training data and utilizing the “limma” package in R, under the standard of P < 0.05 and | log2FC | > 1, 211 DEGs of preeclampsia placenta and normal placenta in the combined dataset were identified. Compared with the normal control group, 155 genes were up-regulated, and 56 genes were down-regulated in preeclampsia placenta. The identified top 100 DEGs are shown in a heatmap and volcano map (**Figures 2A, B**). From the heatmap, it can be observed that the expression of ANKRD37, CRH, LEP, and SIGLEC6 in preeclampsia placenta is significantly increased.

### Functional enrichment analysis of placental DEGs

**Figure 3:**
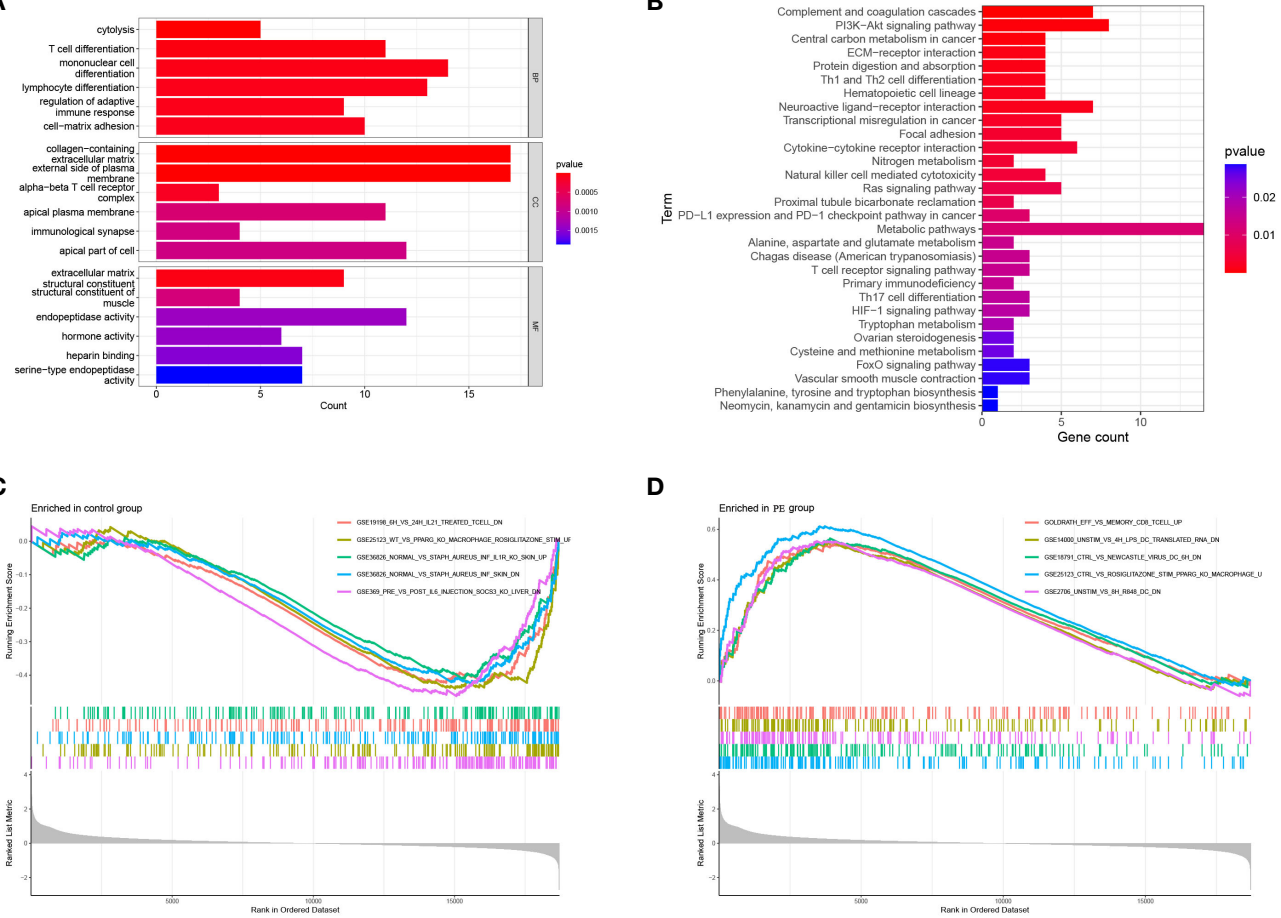
Functional enrichment of DEGs. **(A)** GO analysis: Biological enrichment includes Biological Process (BP), Molecular Function (MF) and Cellular Component (CC); **(B)** KEGG pathway analysis of DEGs; **(C, D)** Enriched Immune Signatures via GSEA.

To further explore the enrichment pathway and function of these 211 DEGs, functional enrichment analysis was carried out using the “ClusterProfiler” in R. According to the GO analysis, these genes were mainly involved in cytolysis, immune cell differentiation, adaptive immune response regulation, and cell-matrix adhesion (BP); extracellular matrix containing collagen, external plasma membrane, and alpha-beta T cell receptor complex (CC); structural constituents of extracellular matrix and muscle (MF) (**Figure 3A**). Additionally, the DEGs were significantly enriched in KEGG pathways, including complement and coagulation cascades, PI3K-Akt signaling pathways, Th1 and Th2 cell differentiation, cytokine-cytokine receptor interactions, and ECM receptor interactions (**Figure 3B**). Furthermore, Gene Set Enrichment Analysis (GSEA) was performed based on the ImmuneSigDB database to evaluate transcriptome changes. A number of immune-related pathways were significantly up-regulated in preeclampsia placentas (**Figures 3C, D**), including the CD8 T cell differentiation pathway gene set (GOLDRATH_EFF_VS_MEMORY_CD8_TCELL_UP), DC activation pathway gene set (GSE14000_UNSTIM_VS_4H_LPS_DC_TRANSLATED_RNA_DN), and macrophage activation gene set (GSE25123_CTRL_VS_ROSIGLITAZONE_STIM_PPARG_KO_MACROPHAGE_UP). There is evidence that immune-related responses play a role in the pathogenesis of preeclampsia.

### Screening biomarkers of preeclampsia using machine learning algorithm

**Figure 4:**
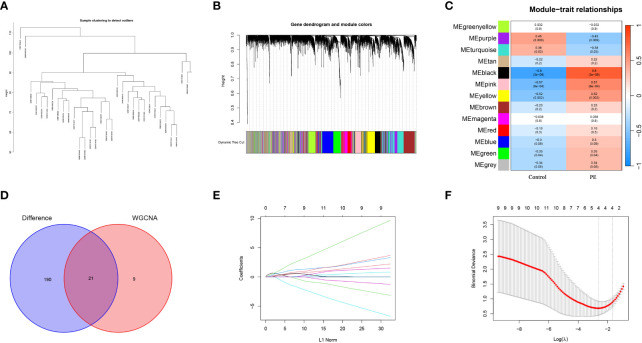
Screening candidate diagnostic biomarkers of preeclampsia by WGCNA and machine learning. **(A)** Cluster analysis is performed based on the correlation between modules to generate an expression cluster analysis diagram illustrating the relationships between modules; **(B)** Merge gene co-expression modules distinguished by various colors in the clustering dendrogram; **(C)** The heatmap illustrates the relationship between modules and traits. The correlation and corresponding p-value for each are located at the intersection of the rows and columns; **(D)** The Venn diagram shows the candidate genes obtained from the intersection of MEblack module of WGCNA and DEGs; **(E)** The curve of partial likelihood deviation versus log(λ) was drawn by using LASSO regression model; **(F)** In lasso model screening, the genes corresponding to the lowest points of the four regression curves are the most suitable candidate genes for preeclampsia.

To identify gene modules related to preeclampsia, a scale-free co-expression network was initially constructed using the R software “WGCNA.” Subsequently, module genes associated with preeclampsia were meticulously screened. For the assessment of sample outliers, the samples were stratified and clustered based on the distribution of sample expression values (**Figure 4A**). Notably, there are slight variations among the samples included in the analysis. In the placental expression profile of preeclampsia, thirteen co-expression modules were discerned, with each color representing a distinct module (**Figure 4B**). Particularly, the MEblack module demonstrated a positive correlation with preeclampsia (r = 0.8, P = 3e-08) (**Figure 4C**). Consequently, it was identified as the key module warranting further research. The intersection of the MEblack module gene and DEGs resulted in 28 intersection genes (**Figure 4D**). The LASSO algorithm was employed to identify potential biomarkers in the placenta of preeclampsia. Utilizing 10-fold cross-validation to select the optimal λ, we identified 4 biomarkers (ANKRD37, CRH, LEP, and SIGLEC6) from the 26 intersecting genes as candidate genes through LASSO analysis (**Figures 4E, F**).

### Construction of biomarker diagnosis model for preeclampsia

**Figure 5:**
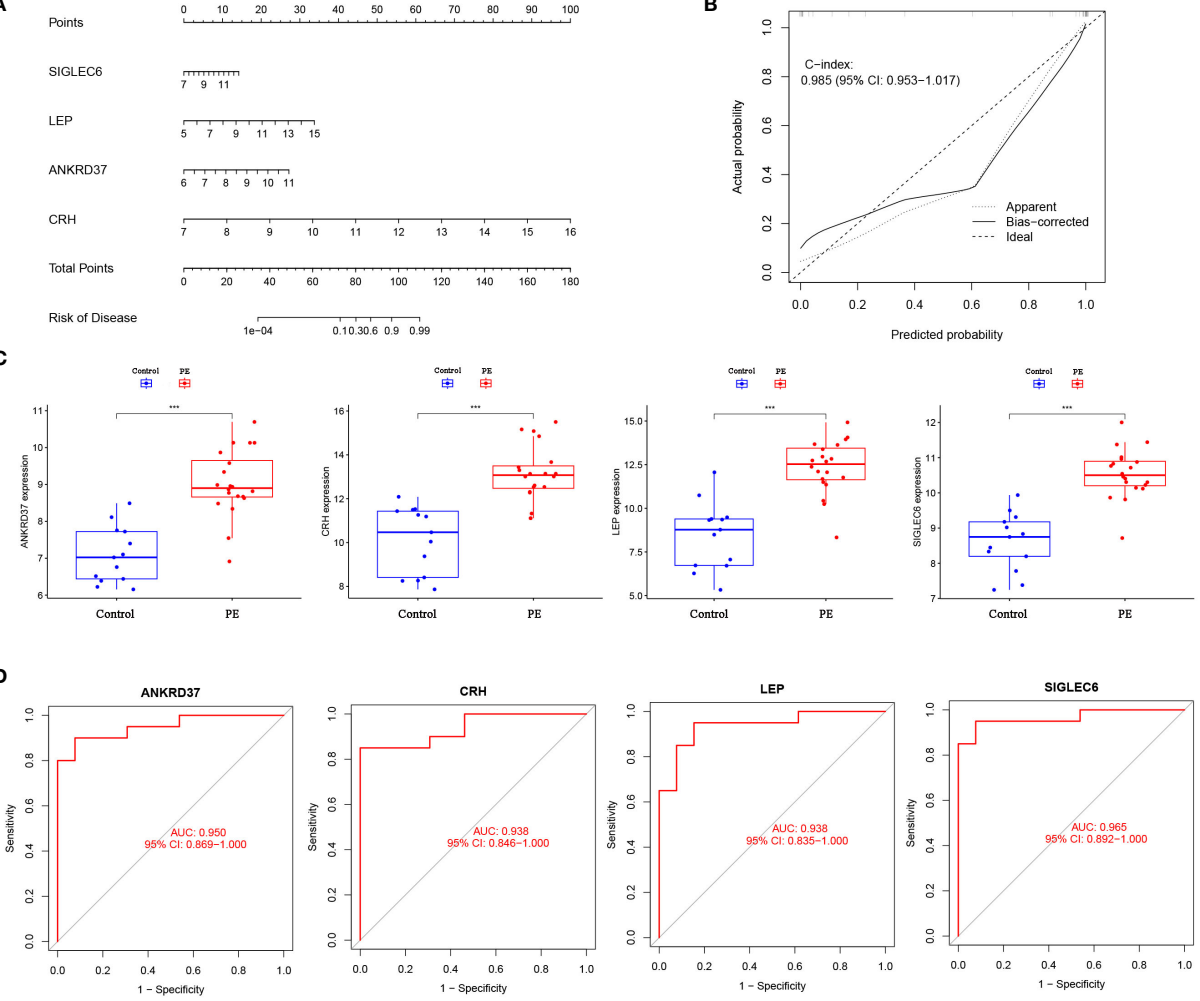
Construction of Nomogram Diagnostic Model for Candidate Biomarkers. **(A)** Nomogram was employed to predict the occurrence of PE. **(B)** Calibration curve was used to validate the consistency of the nomogram. **(C)** Differential expression of candidate genes between preeclampsia group and control group; **(D)** The ROC curve verifies the performance of the lasso regression model; Compared with the control group: ***P<0.001.

In order to further understand the role of screened biomarkers in the diagnosis and prediction of preeclampsia, we based on the nomogram model of four key genes: ANKRD37, CRH, LEP, and SIGLEC6 (**Figure 5A**). Based on the calibration curve (no difference was observed between the predicted value and the observed value), the nomogram model proved that it may have good clinical efficacy (**Figure 5B**). In comparison with the normal control group, the expression levels of the candidate genes ANKRD37, CRH, LEP, and SIGLEC6 in preeclampsia placenta were significantly increased, as visualized by the boxplot function (**Figure 5C**). ROC curves were drawn to evaluate the predictive ability of the LASSO regression, yielding AUC values of 0.933 and 0.833, respectively (**Figure 5D**).

### Evaluation of diagnostic ability of candidate placental biomarkers

**Figure 6:**
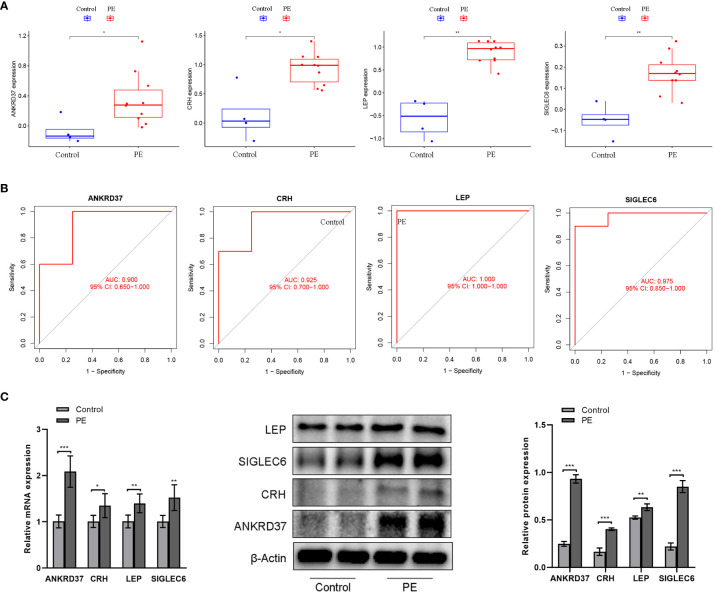
Verification of candidate genes by GSE4707 data set and clinical samples. **(A)** Differential expression of candidate genes between preeclampsia group and control group; **(B)** The ROC curve verifies the performance of the lasso regression model; **(C)** QPCR and western blot were used to detect the expression of characteristic genes in the placenta of patients. Compared with the control group: *P<0.05, **P<0.01.

To further assess the expression patterns and diagnostic utility of a candidate gene identified from the Lasso regression model in the placenta, the GSE4707 dataset was utilized as test data. Expression levels and ROC curves of candidate genes in the placenta of both groups were plotted. Preeclampsia placentas exhibited significantly elevated levels of NKRD37, CRH, LEP, and SIGLEC6 expression compared to the control group (**Figure 6A**). ROC analysis of the GSE4707 dataset further underscored the potential of these biomarkers as promising diagnostic indicators for this condition, with AUC values of 0.933 and 0.833, respectively (**Figure 6B**). Moreover, the results from qPCR and western blot analyses of placental characteristic genes in PE patients aligned with the findings from the computational analysis (**Figure 6C**). These results furnish compelling evidence that NKRD37, CRH, LEP, and SIGLEC6 markers possess the potential to serve as excellent diagnostic biomarkers for preeclampsia.

### Correlation analysis between immune infiltration characteristics of placenta and candidate genes in preeclampsia

**Figure 7:**
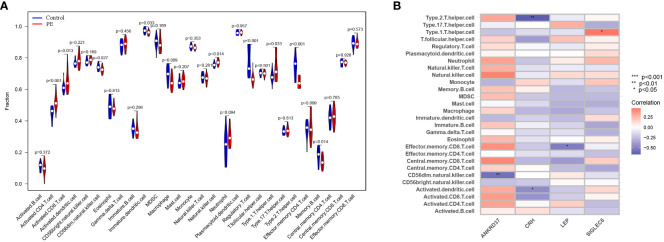
Identification of characteristics of immune cell infiltration in preeclampsia. **(A)** The distribution difference of immune cell types in preeclampsia placenta and control placenta; **(B)** Correlation analysis between candidate genes and immune cell types.

The single-sample gene set enrichment analysis (ssGSEA) scores were used to enrich the gene set related to placental cellular immunity in order to understand the immune function of preeclampsia placentas. The results showed significant differences in the scores of various immune cells between preeclampsia placenta and normal placenta, including Activated.CD4.T.cell, Activated.CD8.T.cell, D56dim.Natural.killer.cell, Immortal.Dendritic.Cell, Macrophage, Natural.Killer.Cell, Regulatory.T.cell, Type.1.T.helper.cell, Type.2.T.helper.cell, and Memory.B.cell (**Figure 7A**). Furthermore, to gain a deeper understanding of the significance of the five candidate genes in placental immunomodulation in preeclampsia, correlation analysis revealed that ANKRD37 is negatively correlated with CD56dim.natural.killer.cell, CRH is negatively correlated with Type.2.T.helper.cell, LEP is negatively correlated with Effector.memory.CD8.T.cell, and SIGLEC6 is positively correlated with Type.1.T.helper.cell (**Figure 7B**). These results underscore the crucial role of ANKRD37, CRH, LEP, and SIGLEC6 candidate genes in placental immunomodulation and immune tolerance.

### The role of candidate genes in placental immune tolerance

**Figure 8:**
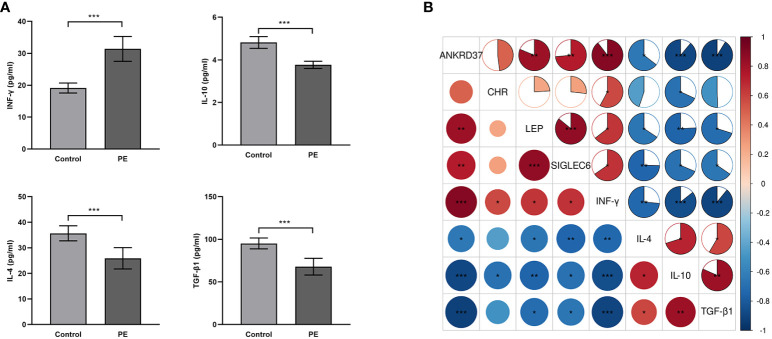
Impact of Candidate Genes on Immune Tolerance-Related Factors in the Placenta of Preeclampsia. **(A)** Assessment of Cytokine Expression Relevant to Placental Immune Tolerance Through ELISA; **(B)** Conducting a pearson correlation test to assess the association between mRNA transcription Levels of candidate genes and the expression of immune tolerance-related factors; Compared with the control group: *P<0.05, **P<0.01, ***P<0.001.

In order to further study the role of key genes in maternal-fetal interface immune tolerance, ELISA was used to evaluate immune tolerance-related factors. We observed an upregulation in interferon-gamma expression and a downregulation in transforming growth factor-beta-1 (TGF-β1), IL-4, and IL-10 in the placenta of patients with preeclampsia (**Figure 8A**). According to correlation analysis, ANKRD37, CRH, LEP, and SIGLEC6 were positively correlated with interferon-gamma and negatively correlated with TGF-0.1 and IL-10, while LEP and IL-10 were negatively associated with LEP, and ANKRD37, CRH, LEP, and SIGLEC6 were positively correlated with interferon-γ(INF-γ) (**Figure 8B**). It is suggested that ANKRD37, CRH, LEP, and SIGLEC6 may contribute to immune tolerance at the maternal-fetal interface by activating or inhibiting the release of immune-related factors.

## Discussion

Preeclampsia, a life-threatening complication in pregnancy characterized by hypertension and organ dysfunction, is intricately linked to placental dysfunction and aberrant immune responses (15, 16). The comprehensive analysis of DEGs in the placenta of preeclampsia patients and normal controls provided crucial insights into the molecular alterations underlying this disorder (17). The identification of 211 DEGs, with 155 up-regulated and 56 down-regulated genes, highlights the significant transcriptional dysregulation in the placental tissue of preeclampsia patients, suggesting the intricate involvement of molecular changes in the pathogenesis of this condition.

Functional enrichment analysis offers crucial insights into biological processes and pathways modulated by these DEGs. Growing evidence suggests that trophoblast necrotizing apoptosis plays a complex role in preeclampsia, involving superficial trophoblast infiltration and inadequate uterine artery remodeling (18). This may be closely related to disorders outside the immune system, caused by abnormal immune system activation and unbalanced differentiation of T cells (19, 20). The GO analysis revealed the enrichment of biological functions related to cytolysis, inflammatory cell differentiation, regulation of adaptive immune response, and cell-matrix adhesion, thereby emphasizing the pivotal role of immune-related processes in placental pathology. Previous studies evaluated the deposition of C5b-9 complement complexes and the exposure of endothelial cells to activated plasma, revealing a significant increase in the deposition of C5b-9 and Von Willebrand factors in the early onset of severe preeclampsia (21). Notably, the enrichment of DEGs in key pathways, including complement and coagulation cascades, PI3K-Akt signaling pathways, Th1 and Th2 cell differentiation, and cytokine-cytokine receptor interactions, underscores the intricate interplay between immune responses and placental dysfunction in preeclampsia.

Further analysis of immune mechanism changes in the placenta of preeclampsia; GSEA findings revealed a significant up-regulation of immune-related pathways, indicating the pivotal involvement of immune responses in preeclampsia pathogenesis. The up-regulation of CD8 T cell differentiation, DC activation, and macrophage activation pathways underscores the active participation of these immune processes in the pathological alterations observed in preeclampsia, suggesting a potential role for immunological dysregulation in the pathophysiology of this disorder. It is reported that preeclampsia may be caused by the decrease of T (Treg) cells specifically regulated by paternal/fetal antigens and the decreased expression of PD-1 on cloned CD8 effector memory T (TEM) cells, resulting in maternal-fetal tolerance (11). Animal experiments have also observed enhanced DC function in mice under “preeclampsia-like” conditions, reflecting local and systemic immune dysfunction in humans (22). Additionally, DAMP molecules released under ischemia and hypoxia, along with STBM binding to TLR, can activate monocytes, DC, NK cells, and neutrophils, thus promoting the persistent inflammatory state of the syndrome (23).

ANKRD37 is a repetitive domain protein associated with anchor proteins, involved in various biological processes, including cell signal transduction, cell cycle regulation, and apoptosis (24). CRH is a peptide synthesized by the hypothalamus, associated with functions such as anxiety, emotion, arousal, feeding, sympathetic activity, and immune and cardiovascular regulation (25). LEP, primarily secreted from adipose tissue, regulates immune, cardiovascular, and metabolic processes (26, 27). SIGLEC6, an immunoglobulin-like domain cell adhesion molecule, plays a crucial role in the immune system. It recognizes and binds glycosylation molecules on bacteria and viruses surfaces, regulating immune responses (28, 29). Clustering based on a scale-free co-expression network and a powerful machine learning algorithm identified potential biomarkers, including ANKRD37, CRH, LEP, and SIGLEC6, emphasizing their potential as candidates for pre-eclampsia diagnosis. The results showed that these candidate genes were significantly up-regulated in the placenta of preeclampsia, highlighting their potential as reliable biomarkers for diagnosis of this disease. The area under the curve (AUC) values in ROC analysis further support their potential diagnostic efficacy in distinguishing preeclampsia patients from normal controls. Studies have shown that ANKRD37 high expression may inhibit the migration and invasion of trophoblast cells through the NF- κ B pathway, potentially contributing to PE development (30). In the hypoxic-ischemic environment of preeclampsia, a novel member of the CRH peptide family significantly increases Ucn2 and Ucn3, further promoting the response to oxidative stress in the placenta (31). LEP, as a pluripotent cytokine, is involved in promoting inflammation, suggesting it may have a crucial relationship with fetal pregnancy outcomes during pregnancy (32). Additionally, increased SIGLEC6 in trophoblast cells impairs vascular endothelial cell function by down-regulating Wnt6/β-catenin signal transduction in preeclampsia (33). However, research on their regulation of immune tolerance in the placenta is still lacking.

The correlation analysis between immunological infiltration characteristics and candidate genes revealed the intricate involvement of ANKRD37, CRH, LEP, and SIGLEC6 in placental immunomodulation. The significant correlation patterns between these candidate genes and specific immune cell subsets, such as natural killer cells, T helper cells, and regulatory T cells, highlight their potential involvement in modulating immune responses at the maternal-fetal interface. This underscores their critical role in preeclampsia immune tolerance and pathology. The maternal immune system needs to tolerate the semi-heterogenic fetus during pregnancy, and this adaptation occurs at the maternal-fetal interface. Failure to tolerate paternal antigens may lead to pregnancy complications, including abortion and preeclampsia (34). On the other hand, in adaptive immunity, the correct increase in regulatory T cells is very critical to ensure immune tolerance to placental cells. A disorder of maternal tolerance can lead to pregnancy complications, having a considerable impact on perinatal morbidity and mortality (35, 36). Evidence shows that CRH may play a key role in the anti-rejection process of implantation and protection of the fetus from the maternal immune system. This is mainly achieved by killing activated T cells through Fas-FasL interaction (37), providing an explanation for the disease.

Th1/Th2/Treg cytokines’ immunopathological effects on pregnancy have been confirmed in animal studies and in human pregnancy (38, 39). Lipopolysaccharide (LPS) was injected into 14.5gd pregnant Wistar rats to induce maternal inflammation and subsequent fetal loss in a dose-dependent manner. Living fetuses also show obvious growth restrictions. Administration of IL-10 and the TNF-α receptor blocker etanercept, with immunomodulatory properties, can prevent pregnancy loss induced by LPS (40). In addition, it is reported that, compared with normal pregnancy, the expression of IL-2 and IFN-γ in the decidua of aborted women is increased, while IL-4 and IL-10 are decreased (38, 41). The analysis of cell subsets in preeclampsia patients found an increase in Th1 and Th17, while decreasing anti-inflammatory subsets Th2 and Treg. This led to a reduction in maternal placenta tolerance (42, 43). The evaluation of ANKRD37, CRH, LEP, and SIGLEC6 in the context of maternal-fetal interface immune tolerance provided crucial insights into their functional significance in modulating immune-related factors. The significant up-regulation of these genes in preeclampsia placenta, as evidenced by qPCR and Western Blot analyses, underscores their potential role in mediating immune tolerance and immune-related factors. The altered expression of immunotolerance-associated factors, including IFN-γ, TGF-β, IL-4, and IL-10, further emphasizes the intricate interplay between these candidate genes and immune responses at the maternal-fetal interface. This underscores their potential contribution to preeclampsia pathological alterations.

**Figure 9:**
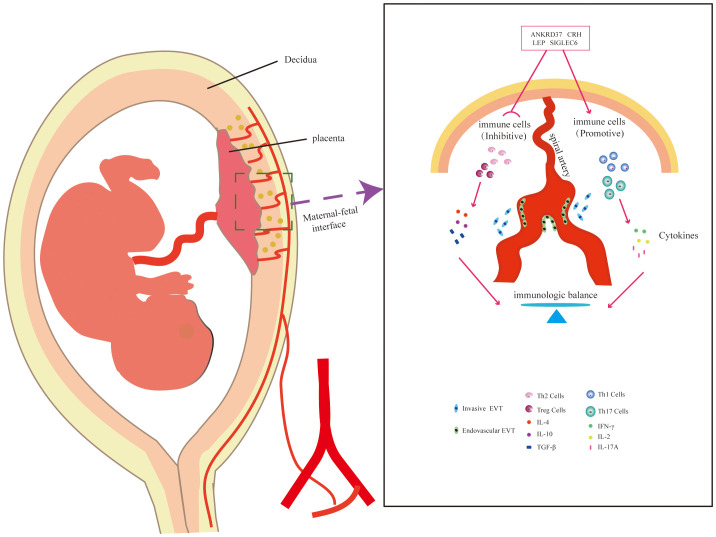
Schematic Representation of the Potential Mechanism of Candidate Genes in Placental Immunomodulation.

In this study, the combined analysis of machine learning and Weighted Gene Co-expression Network Analysis (WGCNA) has provided a distinct advantage in uncovering molecular and functional changes associated with preeclampsia. Leveraging machine learning algorithms has allowed for the efficient analysis of extensive datasets, revealing intricate patterns and aiding in the prioritization of candidate genes for further investigation (**Figure 9**). Although the current research provides key insights into molecular and functional changes related to preeclampsia, several limitations must be considered. The biological verification of candidate genes and their specific roles in immune tolerance and placental pathology requires further *in vitro* and *in vivo* research to clarify their exact roles in the etiology of preeclampsia. Additionally, the clinical applicability and utility of the identified biomarkers must be strictly verified in a larger patient cohort. The findings of this study should be translated into potential clinical applications and therapeutic interventions for preeclampsia.

## Data availability statement

The datasets presented in this study can be found in online repositories. The names of the repository/repositories and accession number(s) can be found below: Gene Expression Omnibus https://www.ncbi.nlm.nih.gov/geo/ (GSE44711, GSE66273, GSE4707) and (Platforms GPL10558, GPL4133, and GPL1708).

## Ethics statement

The studies involving humans were approved by the First Affiliated Hospital of Kunming Medical University (Ethical approval number 31-2, 2022). The studies were conducted in accordance with the local legislation and institutional requirements. The participants provided their written informed consent to participate in this study.

## Author contributions

YM: Conceptualization, Methodology, Visualization, Writing – original draft. XD: Data curation, Methodology, Writing – review & editing. RS: Formal analysis, Software, Visualization, Writing – review & editing. HZ: Data curation, Investigation, Methodology, Writing – review & editing. YQ: Project administration, Writing – review & editing.
