# Supplementary material for: Unveiling immune tolerance pathways in preeclampsia placenta: implications for molecular targets and discovery of potential biomarkers
Source: Front Endocrinol (Lausanne). 2024 Jun 4;15:1385154. doi: 10.3389/fendo.2024.1385154 (PMC11182985; doi:10.3389/fendo.2024.1385154)
Supplement: Supplementary file 1 [file Table_1.docx]

| **Characteristic** | **PE (n=5)** | **Control (n=5)** | ***P* value** |
| --- | --- | --- | --- |
|  | Mean ± SD | |  |
| Age, years | 28.826 ± 3.174 | 29.766 ± 5.281 | 0.743 |
| BMI, kg/m^2^ | 22.745 ± 3.228 | 21.967 ± 2.012 | 0.662 |
| SBP, mmHg | 143.160 ± 2.285 | 126.075 ± 9.220 | 0.0125 |
| DBP, mmHg | 92.712 ± 5.196 | 77.245 ± 3.535 | <0.0001 |
| Diagnosis weeks | 30.335 ± 3.078 | 29.472 ± 3.273 | 0.679 |
| Parity, times | 0.600 ± 0.547 | 0.800 ± 0.836 | 0.668 |
| Gestational, weeks | 38.397 ± 0.210 | 38.819 ± 0.763 | 0.291 |
| Weight, g | 3189.302 ± 449.235 | 3141.868 ± 399.389 | 0.864 |

supplementary table 1 Clinical characteristics of participants
